# Sundown syndrome in patients with Alzheimer’s disease dementia

**DOI:** 10.1590/1980-57642018dn13-040015

**Published:** 2019

**Authors:** Cristiani Sartorio Menegardo, Fernanda Alencar Friggi, Julia Baldon Scardini, Tais Souza Rossi, Thais dos Santos Vieira, Alessandra Tieppo, Renato Lirio Morelato

**Affiliations:** 1 Pós-graduanda em Geriatria pelo Hospital Santa Casa de Misericórdia de Vitória, Escola Superior de Ciências da Santa Casa de Misericórida de Vitória, ES, Brasil.; 2 Estudante de Medicina de Iniciação Científica - PIVIC (Programa de Iniciação Científica Voluntária) pelo Hospital Santa Casa de Misericórdia de Vitória, Escola Superior de Ciências da Santa Casa de Misericórida de Vitória, ES, Brasil.; 3 Mestre em Políticas Públicas e Desenvolvimento Local, Professora Assistente de Geriatria pelo Hospital Santa Casa de Misericórdia de Vitória, Escola Superior de Ciências da Santa Casa de Misericórdia de Vitória, ES, Brasil.; 4 Doutor em Ciências Fisiológicas, Professor Adjunto e Supervisor do programa de Geriatria do Hospital Santa Casa de Misericórdia de Vitória, Escola Superior de Ciências da Santa Casa de Misericórida de Vitória, ES, Brasil.

**Keywords:** Alzheimer’s disease, elderly, neuropsychiatric symptom, sundown syndrome, doença de Alzheimer, idoso, sintoma neuropsiquiátrico, síndrome do entardecer

## Abstract

The sundown syndrome is a complex neurobehavioral disorder in patients with dementia associated with high financial cost and significant caregiver burden. It is a multifactorial phenomenon with unclear pathophysiology, characterized by the presence of neuropsychiatric symptoms in the evening period.

**Objective:**

To analyze the main neuropsychiatric symptoms, their correlation with one another, with comorbidities, and with time of day of greatest symptom intensity in patients with Alzheimer’s disease dementia.

**Methods:**

This is a cross-sectional, observational and explanatory study in which caregivers/relatives of elderly patients with dementia were interviewed using a structured tool called the Neuropsychiatric Inventory (NPI).

**Results:**

The sample studied was composed of 38 patients, 60.5% female and 39.5% male, with mean age of 81±6 (67-94) years. A high frequency of neuropsychiatric symptoms in the evening period was observed, predominantly irritability (55.3%), nocturnal behavior (47.4%), and aggressiveness (42.1%). Only 36.8% of the family caregivers used non-pharmacological strategies.

**Conclusion:**

The frequency of neuropsychiatric symptoms was exacerbated in the evening among patients with Alzheimer’s disease, especially for those behavioral symptoms that had a positive correlation with one another.

## RESUMO

A síndrome do entardecer ou “pôr do sol” - *“sundown syndrome”* - é uma alteração neurocomportamental complexa em pacientes portadores de demência que geram custos financeiros excessivos e desgaste importante a seus cuidadores. Trata-se de um fenômeno multifatorial com fisiopatologia incerta, que caracteriza-se pela presença de sintomas neuropsiquiátricos no período vespertino.

**Objetivo:**

Analisar os principais sintomas neuropsiquiátricos, sua correlação entre si, com comorbidades e o horário do dia de maior intensidade destes em pacientes portadores de demência da doença de Alzheimer.

**Métodos:**

Trata-se de um estudo analítico transversal, observacional e explanatório no qual foram entrevistados cuidadores/familiares de pacientes idosos portadores de demência utilizando uma ferramenta estruturada denominada *Neuropsychiatric Inventory* (NPI).

**Resultados:**

A amostra estudada foi de 38 pacientes, 60,5% feminino e 39,5% masculino, com 81±6 (67-94) anos de idade. Foi observada uma alta frequência de sintomas neuropsiquiátricos no período vespertino, principalmente irritabilidade (55,3%), comportamento noturno (47,4%), agressividade (42,1%). Apenas 36,8% dos cuidadores familiares empregavam estratégias não farmacológicas.

**Conclusão:**

A frequência dos sintomas neuropsiquiátricos são exacerbados ao entardecer em pacientes com doença de Alzheimer; principalmente os sintomas comportamentais com correlação positiva entre si.

In 1941, Cameron first described the presence of neuropsychiatric symptoms in the evening period in elderly patients with neurocognitive decline, which he called *senile nocturnal delirium.* 1 The sundown syndrome is a complex neurobehavioral disorder in patients with dementia that results in high financial costs and significant burden for caregivers.^2^

The cause of sundown syndrome remains unclear. There are no criteria or consensus to define this syndrome, where several conceptual aspects are formulated for a single definition. Some authors restrict the concept to patients with dementia, while others describe the phenomenon for elderly people with intact cognition - despite the low prevalence.^3^ Due to the heterogeneity of the clinical manifestations, some authors limit the definition to neuropsychiatric symptoms, in particular restlessness and irritability, while others include symptoms of anxiety and behavior.^4^

The prevalence of this syndrome in individuals with dementia is estimated to range from 2.4% to 85%^5^ and the pathophysiology is still unclear.^6^^,^ 7 Sundown syndrome is a multifactorial phenomenon, with multiple interacting factors contributing to its occurrence and main phenotypic characteristics. Studies that have attempted to explain the etiology of sundown syndrome can be divided into three major groups: physiological, psychological, and environmental.^8^

Neuropsychiatric symptoms in the evening period and nocturnal onset play an important role in changes to the suprachiasmatic nucleus (SCN), located in the hypothalamus, and to the biological clock of the brain, responsible for circadian cycle alterations.^9^ An important component of circadian rhythm regulation is melatonin, a hormone secreted by the pineal gland in response to darkness, whose production and release is regulated by the SCN itself. Melatonin levels have been shown to decrease during aging and be even further reduced in Alzheimer’s disease (AD) and other neurodegenerative diseases.^10^

In 2005, the American Sleep Disorders Association defined the syndrome as a sleep disorder characterized by nocturnal vagrancy and confusion.^11^ It is characterized by several neuropsychiatric symptoms, most notably: aggressiveness, irritability, delusions, hallucinations, aberrant motor behavior and apathy. This condition is influenced by the circadian cycle, predominating in the late afternoon, early evening and night.^6^^,^ 7

Cohen-Mansfield et al. (2007) evaluated 174 elderly people in 12 long-term care facilities for the elderly in the USA and demonstrated that these symptoms start at approximately 1 pm and reach a higher intensity at around 4 pm.^12^

This study aims to analyze the main neuropsychiatric symptoms, their correlation with each other, with comorbidities and time of day of greatest symptom intensity in patients with dementia due to probable AD. 

## METHODS

A cross-sectional, observational, explanatory study was conducted in which caregivers/relatives of elderly patients with dementia were interviewed. Patients residing in long-term care facilities for the elderly or who had a history of previous or concomitant psychiatric disorders not due to the degenerative process, as well as patients with delirium caused by acute clinical causes, were excluded.

Data on age, gender, education, comorbidities and symptomatic drugs - cognitive symptoms (cholinesterase inhibitors: donepezil, rivastigmine, galantamine and memantine), behavioral symptoms (antipsychotic drugs, anticonvulsants), depression (benzodiazepine and selective serotonin reuptake inhibitors: sertraline, citalopram and trazodone), were taken from medical records or informed by caregivers. 

The non-pharmacological interventions evaluated were those reported in the meetings with caregivers held monthly at the service: stimulation for physical activity, music therapy, adaptation, and organization of the patient’s environment.

The study interventions took place prior to outpatient visits or during the fortnightly meetings with relatives/caregivers of patients with dementia followed at the geriatric medical residency service of the Santa Casa de Misericórdia de Vitória Hospital, Brazil.

For the sample calculation, a total of approximately 1040 patients attending the geriatrics service per year, with 10% having some degree of dementia and considering a sampling error of 10%, test power of 80% (type II error) for a level of significance of 5% (type I error), a sample size of 34 individuals was calculated. Considering 10% of probable loss, a sample of 38 patients was thus determined.

The researchers interviewed the main caregivers of patients with dementia, who were followed up at the service, diagnosed clinically and by imaging (computed tomography or magnetic resonance imaging), who met the criteria for the diagnosis of probable AD according to the Diagnostic and Statistical Manual of Mental Disorders (DSM-IV); and the National Institute of Neurological and Communicative Diseases and Stroke/Alzheimer’s Disease and Related Disorders Association (NINCDS/ADRDA),^13^ and who were able to provide reliable information regarding the neuropsychiatric symptoms presented in the last month. All patients were from the Brazilian Unified Healthcare System. They reported family income of up to two minimum wages and 95% had low educational background (up to 4 years of schooling). Disease time was counted from the onset of cognitive and behavioral symptoms reported by relatives.

Sundown syndrome was used to describe a wide range of neuropsychiatric symptoms occurring in individuals with dementia. We used the NPI (Neuropsychiatric Inventory), a reliable instrument, validated in Brazil,^14^ consisting of a questionnaire, administered to the family member and/or caregiver, composed of structured questions regarding the intensity and frequency of neuropsychiatric symptoms observed in the past month in patients with a diagnosis of dementia. Twelve symptoms were assessed: delusions, hallucinations, restlessness, depression, anxiety, euphoria, apathy, disinhibition, irritability, aberrant motor activity, nocturnal behavior disorders and appetite changes.^14^ In addition, the caregivers/domains were questioned about the time of onset time and of greatest intensity of these symptoms. Caregiver burden was not assessed in this study.

The following instruments were used: the Clinical Dementia Rating (CDR) scale and Mini-Mental State Examination (MMSE). The CDR scale is an instrument that determines the stage of functional impairment of dementia.^15^ The final classification of the CDR is obtained by analyzing the classifications by category, according to a set of validated rules, classified as normal (CDR = 0), borderline (CDR = 0.5), mild dementia (CDR = 1), moderate (CDR = 2) or severe dementia (CDR = 3).^16^^,^ 17

The MMSE evaluates cognitive status. This instrument’s maximum score is 30, with cut-off points according to educational level. It addresses questions about memory, attention, orientation, language and visuospatial skills.^18^ The median values for educational groups were: 20 for illiterates; 25 for 1 to 4 yrs; 26.5 for 5 to 8 yrs; 28 for 9 to 11 yrs and 29 for higher levels.^19^

The dependent variable (outcome) was “SUNDOWN SYNDROME”: defined as exacerbation of restlessness (neuropsychiatric) symptoms in the afternoon and evening periods.^7^ The independent variables were: Alzheimer’s disease dementia, stage of dementia (CDR), caregiver’s degree of kinship, associated chronic non-communicable diseases (diabetes, hypertension and hypothyroidism) and drugs in regular use for Alzheimer’s disease.

The data were analyzed using descriptive statistics with continuous and categorical variables. The continuous variables were expressed as mean and standard deviation, whereas the categorical variables were expressed as percentages. Spearman correlation (r_s_) was applied to determined correlation between variables. SPSS 25 software, licensed to the EMESCAM (Escola Superior de Ciências da Santa Casa de Misericórida, Vitória, Brazil), was used to analyze the data. Values less than 0.05 were considered significant. The patient’s relative and/or caregiver signed the Free and Informed Consent Form. The project was approved by the CEP-EMESCAM under permit CAAE 68149917.0.0000.5065 (June 5^th^, 2017).

## RESULTS

Initially, 56 patients of both sexes, with dementia were included in the geriatrics care service. Eighteen (18) patients were subsequently excluded because they presented non-Alzheimer’s disease dementia (4), vascular dementia (6) and mixed dementia (8), giving 38 patients who met the criteria for the diagnosis of probable AD, according to NINCDS/ADRDA criteria. 

The study sample consisted of 38 patients, 60.5% female and 39.5% male, aged 81±6 (67-94) years, diagnosed with Alzheimer’s disease for an average of 4.6 years (1-11 years), predominantly (89.5%) in the moderate phase of the disease (CDR 2). The mean MMSE score was 13 (±5). Regarding associated comorbidities, 63.2% were hypertensive and 23.7% had diabetes mellitus; 39.5% of patients, in addition to Alzheimer’s disease dementia, had two or more other comorbidities (multimorbidity), as shown in Table 1.

**Table 1:** Characteristics of sample with Alzheimer's disease.

Age (years)		81±6 (67-94)
Gender		60.5% female/39.5% male
Education (years)		3.15±1.9 (0-8)
MMSE (0-30)		13±5 (5-22)
CDR	1	5.3%
	2	89.5%
	3	5.3%
Arterial hypertension		63.2%
Diabetes mellitus		23.7%
Hypothyroidism		7.9%
Anticholinesterases		65.2%
Atypical neuroleptics		63.2%
Serotonin reuptake inhibitors		34.2%
Benzodiazepines		30.4%
Memantine		13.2%
Non-pharmacological treatment		36.8%

In 73.7% of patients, caregiving was performed by the daughter, in 5.3% by the spouse, and in only 2.6% by a formal caregiver, because the patient sample comprised low-income users of the public healthcare network. 

Symptom onset time was more frequently in the early afternoon (1 pm) and highest symptom intensity occurred in the late afternoon (4-5 pm) for 60.5% (23) of patients. The most frequent neuropsychiatric symptoms were: irritability (55.3%), nocturnal behavior (47.4%), aggressiveness/agitation (42.1%), hallucinations (39.5%), aberrant motor behavior (36.8%) and apathy (Table 2). A total of 55.3% (21 patients) presented symptoms every day of the week, while 23.7% (9 patients) exhibited them 4 to 5 times a week.

**Table 2:** Neuropsychiatric symptoms presented in Sundown Syndrome.

Symptom	Number of patients	%
Irritability	21	55.3
Nocturnal behavior	18	47.4
Aggressiveness	16	42.1
Hallucinations	15	39.5
Aberrant motor behavior	14	36.8
Apathy	14	36.8
Disinhibition	10	26.3
Anxiety	9	23.7
Delusion	7	18.4
Euphoria	6	15.8
Eating alterations	6	15.8

Correlations between symptoms and symptoms, as well as between symptoms and comorbidities are shown in Table 3. 

**Table 3:** Correlation between symptoms and symptoms, as well as between symptoms and comorbidities.

Symptom	Symptom and comorbidities	
	Positive correlation	Negative correlation
Irritability	Aberrant motor behavior (r_s_ + 0.384; p=0.01) Diabetes mellitus (r_s_ + 0.302; P= 0.03)	-
Nocturnal behavior	Eating Alteration (r_s_ + 0.337, p=0.04)	-
Aggressiveness	-	Apathy (r_s_ - 0.345; p=0.03)
Hallucinations	Delusions (r_s_ + 0.636; p<0.001) Disinhibition (r_s_ + 0.489; p=0.002)	-
Apathy	-	Eating Alterations (r_s_ - 0.434; p=0.007) Aggressiveness (r_s_ - 0.434; p=0.03)
Anxiety	Eating Alterations (r_s_ + 0.522; p=0.001)	Disinhibition (r_s_ - 0.320; p=0.05)
Delusion	Hallucinations (r_s_ + 0.636; p<0.001) Disinhibition (r_s_ + 0.636; p=0.001)	-
Eating alterations	Nocturnal behavior (r_s_ + 0.337; p=0.04) Anxiety (r_s_ + 0.552; p=0.001)	Apathy (r_s_ - 0.434; p<0.001),
Aberrant motor behavior	Irritability (r_s_ + 0.384; p=0.01)	-
Disinhibition	Anxiety (r_s_ + 0.489; p=0.02)	-
Euphoria and depression	-	-

Spearman Correlation (r_s_), positive (+) and negative (-), and significance (r) < 0.05.

A total of 63.2% of caregivers, for lack of knowledge, did not use non-pharmacological strategies to reduce neuropsychiatric symptoms, whereas 36.8% applied knowledge acquired during regular service meetings to ease these symptoms.

Although anticholinesterases are recommended, the pharmacological classes used for symptom treatment were: anticholinesterases (65.2%), atypical neuroleptics (63.2%), serotonin reuptake inhibitors (34.2%), benzodiazepines (30.4%) and *memantine* (13.2%).

## DISCUSSION

Results of the Neuropsychiatric Inventory (NPI) applied to caregivers/relatives of patients diagnosed with probable Alzheimer’s disease revealed that most patients presented symptoms in the early evening, with highest intensity between 4 and 5 pm. Ferrazzoli et al. (2013) observed the same symptom intensity in the evening period and identified this as a possible biomarker of frailty in patients with AD.^20^

The main symptoms observed were irritability, nocturnal behavior, aggressiveness, hallucinations, aberrant motor behavior and apathy. We observed an association between irritability and aggressiveness and nocturnal behavior. Most patients (55.3%) had neuropsychiatric symptoms every day of the week. Bremenkamp et al. (2014) observed these same three neuropsychiatric symptoms as the main causes of psychological distress among caregivers.^2^

The correlation between neuropsychiatric symptoms showed that euphoria and depression did not exhibit a significant correlation with other symptoms. These multiple correlations between symptoms are expected because, in clinical practice, the concomitant presence of more than one neuropsychiatric symptom is common.^21^

An earlier study performed in our service, found a positive correlation between neuropsychiatric symptoms, particularly aberrant motor behavior and irritability.^2^

Of the comorbidities and symptoms observed, only diabetes mellitus correlated with irritability. Hypertension and hypothyroidism showed no correlation with the symptoms assessed.

Most patients were female, of advanced age, with disease diagnosed for at least four years and at a moderate stage (CDR 2) when presenting a higher frequency of neuropsychiatric symptoms. As a result of advanced age, a high frequency of associated comorbidities was observed.

Caregiving was performed mostly by daughters (73.7%), similar to previous studies.^2^^,^ 22

Some caregivers (36.8%) used non-pharmacological strategies, drawing on knowledge acquired at a meetings about Alzheimer’s disease held in the service. Javdpour et al. (2009) demonstrated that participation in groups is very cost-effective for assisting caregivers and patients with dementia.^23^

The main pharmacological classes employed were anticholinesterases. Although this is the recommended treatment for moderate forms of dementia, there is no evidence of its benefit in the treatment of neuropsychiatric disorders, including sundown syndrome.^7^ Atypical neuroleptics were the second class observed, although these are the most used drugs in the treatment of the neuropsychiatric symptoms of dementia, studies are conflicting concerning the decrease in evening symptoms of sundown syndrome.^7^

This study had some limitations, such as the small sample size, as well as the difficulty obtaining accurate data about some patients, since in some interviews, information was not obtained from the main caregivers, but from family members present at the time, and was complemented with data from medical records. Caregiver stress - one of the components of the NPI, was not evaluated. However, in a previous study, agitation/aggressiveness proved the main stressful symptoms for family members.^2^

In conclusion, there are frequent neuropsychiatric symptoms exacerbated in the evening time, which could be mitigated if there was previous knowledge of their non-intentionality. These findings show the importance of knowledge of this syndrome by professionals and caregivers of patients with Alzheimer’s disease dementia. 
